# Pithos – a scalable and secure data container for FAIR-compliant research data management in life sciences

**DOI:** 10.1515/jib-2025-0051

**Published:** 2026-08-12

**Authors:** Jannis Schlegel, Sebastian Beyvers, Maria Hansen, Lukas Brehm, Alexander Goesmann, Frank Förster

**Affiliations:** Bioinformatics and Systems Biology, Justus Liebig University Giessen, 35390, Giessen, Germany; Bioinformatics Core Facility, Justus Liebig University Giessen, 35390, Giessen, Germany

**Keywords:** research data management, data container, data protection, integrative bioinformatics, Pithos

## Abstract

Modern research techniques have led to exponential growth in the volume and complexity of scientific data. Consequently, managing these volumes securely and efficiently has become a major challenge. While all research domains face these challenges, life science research is particularly affected because current approaches often rely on a large set of different file formats, with metadata stored in separated databases or spreadsheets. This leads to fragmented datasets, orphaned data, and compromised research reproducibility. Traditional solutions also force researchers to choose between security and accessibility, with encrypted files preventing selective access and indexed formats lacking adequate security for sensitive data. These limitations are particularly problematic in large-scale genomic studies where researchers must decompress multi-gigabyte files to access specific regions, creating computational bottlenecks and inefficient network usage when working with cloud-stored datasets. We introduce Pithos, a next-generation file format specifically designed for scientific data management in distributed cloud environments. The format uses content-defined chunking to enable efficient deduplication across distributed storage systems, thereby reducing storage costs and bandwidth requirements. The append-only structure ensures data immutability and allows for incremental updates without compromising content. Benchmark results show that Pithos outperforms existing solutions in read and write performance, with comparable or improved storage efficiency.

## 1 Introduction

Life science researchers using modern experimental techniques face fundamental challenges in managing the substantial amounts of data generated [1]. Current bioinformatics data management approaches typically rely on collections of disparate file formats, each optimized for specific use cases. Genomic data is often stored in FASTQ, BAM, or VCF formats [2], while the associated metadata is stored in different databases or spreadsheet files [3]. This fragmentation makes it difficult to discover and reuse the data [3]. The separation of data from metadata often results in orphaned datasets, causing the loss of crucial experimental context and undermining the reproducibility of research findings. Additionally, current solutions often force researchers to choose between security and accessibility. Encrypted files sacrifice the ability to perform selective data access, and indexed formats lack adequate security measures for sensitive genomic information [4].

The limitations of traditional approaches are particularly apparent in large-scale genomic studies and multi-omics projects. Researchers frequently need to decompress multi-gigabyte files to access specific genomic regions, creating computational bottlenecks that limit interactive analysis capabilities [5]. These large-scale use cases often rely on object storage systems like S3, which due to their unique characteristics make accessing specific parts of large, compressed collections of files more difficult. This results in inefficient network usage and increased latency when accessing cloud-stored datasets [6].

These technical challenges are further complicated by the need to implement the FAIR principles that require data to be findable, accessible, interoperable, and reusable [7].

Implementing the FAIR principles enables communities to accelerate scientific discovery, reduce redundant data collection efforts and facilitate more robust and reproducible research that builds systematically on previous work. Emerging approaches like Zarr for multidimensional array data or RO-Crate (Research Object Crate) as an integrated view on a Research Object offer promising frameworks for packaging research data with standardized metadata and establishing clearer provenance chains [8]. However, they do not clearly address how to integrate with sensitive and encrypted data, and they often lack the capability to effectively search large, encrypted, and compressed datasets.

Here we present Pithos, a novel package file format specifically designed to enhance research data stored in object storage, with particular emphasis on bioinformatics applications. Named after a large ancient Greek storage container, Pithos addresses multifaceted challenges through an integrated design that combines security, performance, and metadata management within a single, coherent container file format. Instead of treating encryption, compression, and indexing as separate issues, our format unifies these capabilities in a coordinated manner that retains the benefits of each while overcoming their traditional limitations. As a container format it does not replace existing file formats but integrates any format similar to traditional archive formats like tar or zip.

## 2 Related works

The development of Pithos builds upon several established technologies and standards that address different aspects of data packaging, compression, and security in scientific data management.

The Unix tar archiving format (tar) has served as the foundation for data aggregation across diverse computing environments for decades, providing a simple mechanism to bundle multiple files into a single container while preserving file metadata and directory structures. However, tar’s sequential access model requires reading through entire archives to locate specific files, making it unsuitable for random access patterns common in large-scale genomic analysis. Additionally, tar lacks native support for compression and encryption, forcing users to rely on external tools that create additional processing overhead and complicate data access workflows.

BagIt and its evolution into Datacrates represent significant advances in research data packaging by introducing standardized approaches to data integrity verification and provenance tracking. BagIt establishes a lightweight specification for creating “bags” of digital content with embedded checksums and metadata, ensuring data authenticity during transfer and storage operations. Datacrates extend this concept by providing structured metadata frameworks that support richer descriptions of research objects and their relationships. While these approaches excel at maintaining data integrity and provenance, they primarily focus on metadata organization rather than addressing performance challenges associated with large-scale data access, compression efficiency, or security requirements for sensitive datasets.

The Zstandard (Zstd) [9] compression algorithm offers superior compression ratios and decompression speeds compared to traditional algorithms like gzip, making it particularly attractive for scientific data processing workflows. Zstd’s tunable compression levels allow users to balance storage efficiency against processing time, while its streaming capabilities support incremental compression of large datasets. However, when applied independently, Zstd compression prevents random access to specific portions of compressed data without full decompression, limiting its utility for interactive analysis of large genomic files where researchers frequently need to access specific chromosomal regions or individual samples.

Crypt4GH is a specialised encryption standard designed specifically for sharing sensitive biological data, created by the genomics community. It employs modern cryptographic techniques, including ChaCha20-Poly1305 [10] authenticated encryption, and supports sophisticated key management scenarios in which multiple recipients can be granted access to specific portions of encrypted datasets. Crypt4GH’s header-based key distribution mechanism enables the fine-grained access control that is essential for collaborative research involving sensitive human genomic data. However, despite these security advantages, Crypt4GH does not address compression optimization or provide multifile support. This means that additional tools and processing steps are required to achieve efficient storage and query performance for large datasets.

RO-Crate (Research Object Crate) provides a comprehensive framework for packaging research data with rich metadata descriptions based on schema.org vocabularies and JSON-LD serialization. This approach enables semantic annotation of research objects, facilitating automated discovery and integration across heterogeneous data repositories. RO-Crate’s emphasis on FAIR principles and interoperability makes it valuable for creating self-describing research datasets that can be understood and reused by both humans and machines. However, RO-Crate focuses primarily on metadata standardization and does not specify mechanisms for handling encrypted data, optimizing storage efficiency, or enabling high-performance access patterns required for large-scale computational genomics workflows.

While each of these technologies addresses important aspects of scientific data management, they typically operate as independent layers that must be composed together, often resulting in suboptimal performance characteristics and increased complexity in data processing pipelines. The integration challenges become particularly apparent when dealing with sensitive genomic datasets that simultaneously require encryption for regulatory compliance, compression for storage efficiency, random access for interactive analysis, and rich metadata for reproducibility and discovery.

The Multi Layer Archive (MLA) [11] project represents the most recent development with this integrative approach to create a secure and versatile archive format, combining encryption, compression and random access to individual files inside the container. However, MLA lacks mechanisms for block-level deduplication that could optimize storage for datasets with repetitive sequences, and its access control model does not support fine-grained permissions where different recipients can access specific subsets of files within a single archive. Additionally, MLA does not provide integrated support for associating rich scientific metadata directly within the archive structure, requiring external mechanisms to associate experimental context and semantic annotations.

## 3 Pithos

Cloud storage systems excel at handling massive datasets, but accessing specific portions of large encrypted and compressed archives remains problematic. Traditional archive formats force complete decompression and decryption to reach individual files, creating substantial performance penalties when users need only small subsets of data.

Pithos represents a comprehensive solution that brings together the strengths of existing technologies while addressing their shared limitations through unified design principles. Rather than layering separate tools for compression, encryption, and metadata management, Pithos integrates these capabilities within a single container format that maintains performance, security, and semantic richness simultaneously.

The format is designed to operate efficiently in cloud-native environments where object storage systems dominate, while providing the granular access control and metadata integration essential for collaborative genomics research. By treating data blocks as content-addressable units, Pithos enables deduplication and selective access patterns that traditional archive formats cannot support.

The following sections detail how Pithos achieves this integration through its structural design and specialized processing mechanisms.

### 3.1 Architecture

**Figure 1:**
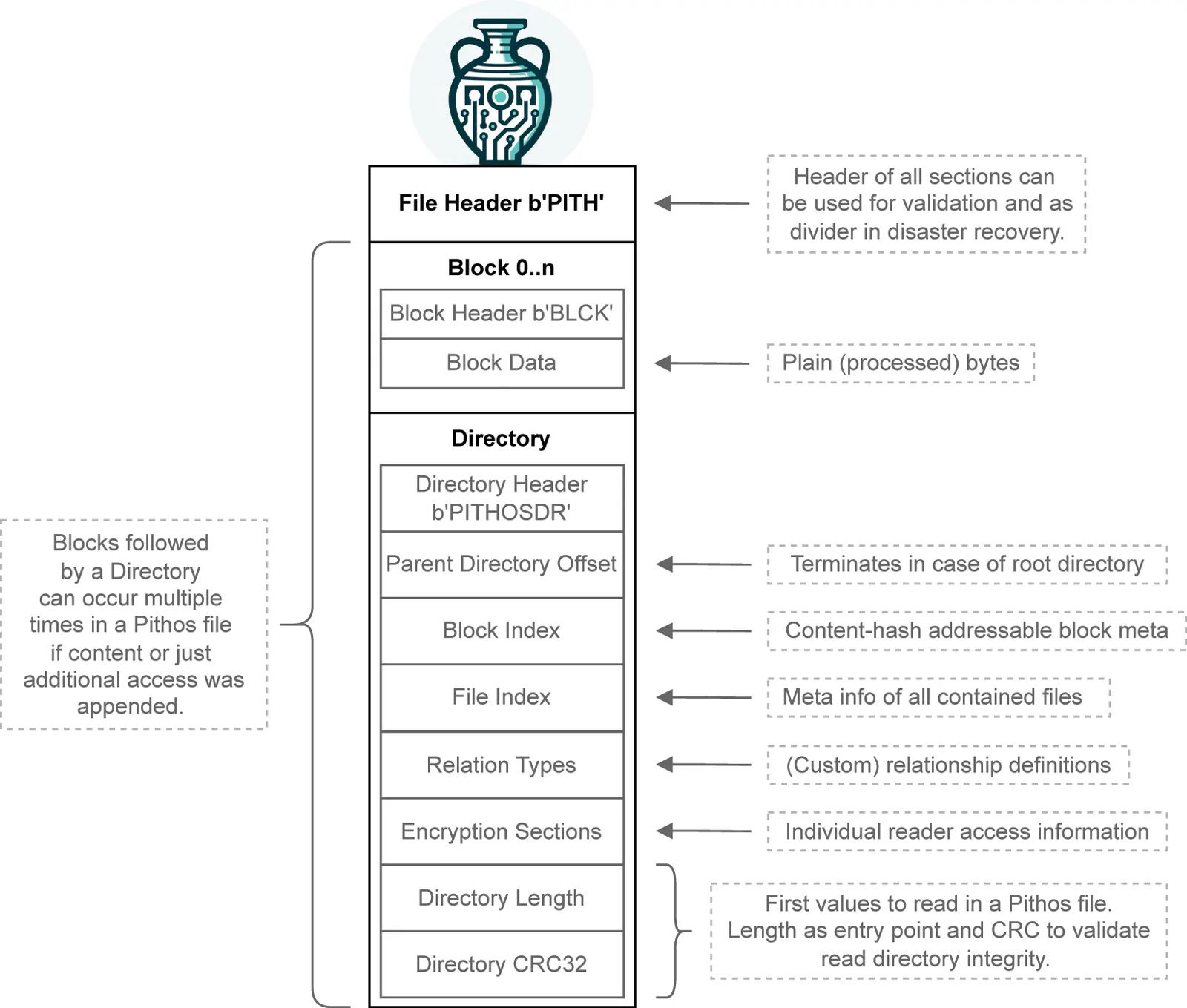
Schematic illustration of the hierarchical organization of a Pithos file. A Pithos file consists of a header (“PITH”), a sequence of processed data blocks (“BLCK”), and an append-only directory section (“PITHOSDR”). The directory catalogs blocks and files with hashes, offsets, metadata, and relationships, supports deduplication, and may contain multiple encrypted sections for fine-grained access control. Each directory links to its predecessor, forming an immutable audit trail, and ends with a length field and CRC32 checksum to ensure integrity.

The Pithos architecture prioritizes simplicity while maintaining robustness, following a deliberate structural approach that ensures straightforward and reliable parsing even under adverse conditions (Figure 1). Each structural component is preceded by a minimal header which acts as a marker to validate the start of a component.

Every Pithos file begins with a file header containing the magic bytes (“PITH”) and a version identifier, establishing format identity and compatibility requirements.

Following the file header, the format consists of a section of processed data blocks, each of which is preceded by a minimal header (“BLCK”). These data blocks contain the actual file content after it has been processed through compression and encryption.

Every Pithos file must end with a directory section marked with the bytes (“PITHOSDR”), which serves as the authoritative catalog for the entire archive. When previous directories exist in the file, the current directory includes a backwards-chaining reference to the preceding directory region, creating an immutable audit trail that supports the append-only architecture. This reference allows implementations to trace the complete evolution of the archive’s contents across multiple append operations.

Within each directory, an encrypted or unencrypted index catalogs all data blocks with their BLAKE3 hashes [12], precise offset information, stored sizes, and processing flags indicating compression levels and encryption status. This content-addressed approach enables automatic deduplication of identical data blocks regardless of their position within different files in the container. Alongside the block index, the directory maintains a comprehensive index of all contained files, including their technical metadata such as permissions, creation and modification timestamps, file types, and relationships to other files within the archive. A descriptive list of all defined relations within the container is also included in the directory, establishing the possibility for semantic connections between files through standardized relationship types.

Multiple individual encryption sections accommodate complex access control scenarios where different parties require access to different subsets of archive content. Each encryption section uses X25519 public key cryptography [13] to securely distribute access keys to authorized recipients while maintaining privacy about other authorized parties.

To ensure data integrity and enable validation, each directory concludes with its length in bytes followed by a CRC32 checksum [14] covering all preceding directory fields. This checksum excludes itself from the calculation, providing a reliable mechanism for detecting corruption in the critical metadata structures.

The append-only architecture also implies that both data blocks and directory sections may occur multiple times within a single file as content is added over time. Each append operation adds new data blocks followed by an updated directory that only contains the new additions and a reference to the preceding directory. This design pattern enables safe concurrent access and provides versioning capabilities.

### 3.2 Smart compression

The achievable compression rate varies greatly depending on the data format and the specific composition of the data in each case. Thus, sequences quality scores compress differently than nucleotide sequences, and both behave differently from annotation data. Traditional formats apply uniform compression settings that waste resources on incompressible data or miss savings opportunities. Pithos implements smart compression probing that evaluates each block independently, storing compression levels in processing flags ranging from level 0 (no compression) through level 7 (maximum compression). Zstd’s exceptional speed and compression ratio make it therefore ideal for genomic data processing, where high-entropy data chunks remain uncompressed while repetitive sequences achieve substantial size reductions. This approach optimizes storage costs and processing time in sequencing pipelines while maintaining the fast decompression speeds essential for real-time analysis workflows.

### 3.3 Data chunking and deduplication

Pithos’s content-addressed deduplication operates at the block level by using the FastCDC [15] implementation of the content-defined chunking method which has configurable minimum, average, and maximum chunk size values. Each chunk is identified by its content BLAKE3 hash, providing detection and elimination of redundant data throughout the archive, regardless of file or position. This process further reduces cloud storage costs and optimizes bandwidth utilization when managing large genomic datasets and bioinformatics workflows that often contain overlapping sequence data across multiple analyses.

### 3.4 Granular security

Genomic research can require individual access control where different collaborators need access to different data subsets. On the block-level Pithos implements convergent encryption using SHAKE256 [16] for deterministic key derivation. The association of the specific blocks a file is composed of is additionally encrypted by a randomly generated X25519 key which is then distributed to recipients in the encryption sections as needed. The recipient data is finally encrypted with a shared key obtained via X25519 key exchange, which allows both sides to access the files stored in the recipient data. This enables selective data sharing where collaborators access only authorized subsets while maintaining cryptographic separation. For example clinical researchers can access patient samples while genomics analysts access only de-identified sequence data from the same archive, preventing unauthorized cross-access.

### 3.5 Random access on compressed and encrypted data

However, for data to be processed effectively, the container format must also allow quick access to the encrypted and compressed data. Pithos provides efficient random access to file ranges through its content-defined chunking system in combination with the directory, which maintains comprehensive metadata for each block, including byte offsets, original and stored sizes, and processing state. As with this each block can be individually decompressed and decrypted as needed, arbitrary data in a Pithos container can be partially accessed by just extracting the blocks which cover the specific requested file or range. This capability is particularly valuable in integrative bioinformatics workflows where researchers need to query specific genomic regions, extract subsets of large datasets, or perform targeted analysis on massive multi-omics files without downloading and decompressing entire archives.

### 3.6 Metadata integration

The management of research metadata involves practical trade-offs that vary in severity depending on the organizational strategy used. External metadata systems offer advantages for lifecycles with continuous curation, versioning, and enrichment, allowing metadata to evolve independently of immutable raw data. But maintaining a persistent link between data and metadata comes with the cost of requiring robust identifier systems and continuous infrastructure. When such linkage is lost, whether through link rot, service discontinuation, or infrastructure migration, data interpretability may be compromised. Pithos addresses this challenge by offering support for diverse metadata management strategies. The format embeds semantic metadata directly, treating it like regular files but distinguishing it with metadata type indicators. Data files maintain relationship references to associated metadata, supporting predefined standard links such as ”describes”, ”annotates,” and ”derived from” as well as custom relationships. The embedded metadata capabilities of Pithos can easily coexist with external metadata systems in various ways. If no external metadata system is available Pithos itself can be used for the metadata management. Otherwise, Pithos can serve as as anything from a complete mirror of the external metadata systems state down to providing just a stable baseline of essential metadata. This baseline ensures that data records are self-contained and remain interpretable even in cases of complete failure of external metadata infrastructure. This is particularly valuable for distributed environments where network connectivity or service availability cannot be guaranteed even when the embedded metadata may not always be completely up to date.

## 4 Results

Pithos is at least on-par with the feature set of other tools, as it supports all necessary features which is required for modern data management (Table 1).

**Table 1:** Comparison of key features across data storage and archival solutions. Pithos uniquely offers configurable content-defined chunking with deduplication, while traditional formats like tar+gzip only provide basic compression without encryption or random access capabilities. HDF5 [17] also supports random access and metadata support but lacks encryption, whereas Crypt4GH prioritizes security but does not provide compression options.

Feature	Pithos	MLA	HDF5	tar+gzip	Crypt4GH
Data chunking	Configurable content-defined chunking	Fixed 4MiB	Configurable	No	Fixed 64KiB
Deduplication	Based on block hash	No	No	No	No
Compression	Smart per-block (Zstd)	Block-level (Brotli)	Block-level (native gzip, szip, lzf)	gzip only	No
Encryption	ChaCha20Poly1305 + X25519	AES-256-GCM + X25519/ML-KEM	No	No	ChaCha20Poly1305 + X25519
Random access	Block-level with index	Seekable chunks	Full random access with index	Sequential only	Block-level
Metadata support	Embedded metadata files	No structured metadata	Attributes on groups and datasets	File attributes only	No
Custom metadata schemas	Any format as file content	No	Compound attribute types	Extended headers	No
Multifile support	Hierarchical directory structure	Native with system entry	Hierarchical groups native (archive)	No	

The write performance evaluation (Table 2) used four categories of datasets to assess the behavior across different file size distributions and directory structures. For the single large file test, the GRCh38.p14 human reference genome (3.2 GiB in FASTA format) was used as a representative large, homogeneous file, which is typical of genomic reference data. For scenarios involving the storage of multiple versions of large files, two versions of the GRCh38.p14 reference genome were used: GCF_000001405.40 and GCA_000001405.29, both 3.2 GiB. A test configuration with a more complex directory structure including heterogeneous files utilized the nf-core [18] atacseq pipeline test profile output containing 748 heterogeneous files distributed across 56 directories, simulating realistic bioinformatics pipeline outputs with varied file types and sizes. Finally, the many small files scenario employed a collection of 1 14 ,733 files containing short Internal Transcribed Spacer 2 (ITS2) [19] sequences, representing high file-count scenarios common in sequence databases.

**Table 2:** Write performance statistics comparison in seconds.

Metric		Pithos	MLA	HDF5	tar+gzip
Single large file	Mean	27.769	169.391	26.809	385.840
	Median	27.830	168.528	26.539	387.796
	StdDev	0.921	21.304	1.063	8.603
Large file versions	Mean	37.583	305.419	49.490	754.766
	Median	38.290	294.923	48.789	748.741
	StdDev	3.320	46.865	1.176	15.345
Complex directory structure	Mean	1.907	4.745	1.976	7.888
	Median	1.879	4.741	1.708	7.682
	StdDev	0.093	0.036	0.649	0.635
Many small files	Mean	58.847	3.891	43.085	3.603
	Median	59.215	2.339	42.632	3.448
	StdDev	6.012	0.065	2.140	0.409

Pithos was configured with FastCDC chunking using minimum, average, and maximum chunk sizes of 1 MiB, 4 MiB and 16 MiB respectively, with Zstd level 4 compression and encryption for a single recipient. MLA used its 4 MiB chunks with Brotli compression and encryption for a single recipient. HDF5 utilized 4 MiB chunk size also with Zstd level 4 compression, though no encryption was applied due to format limitations. The tar+gzip baseline performed sequential archiving without chunking, using default gzip compression settings and no encryption capability. All measurements were conducted using identical hardware and storage configurations to ensure comparative validity across solutions.

The read performance evaluation (Table 3) assessed three distinct access patterns to evaluate storage solution efficiency across different retrieval scenarios. The first test measured full file extraction performance by reading the complete GRCh38.p14 reference genome back to disk from the archived format. The second test evaluated partial file access capabilities by extracting a specific genomic region corresponding to Chromosome 13 primary assembly from the archived reference genome, simulating targeted data retrieval common in genomic analysis workflows. The third test assessed random access performance for small file collections by retrieving a randomly selected subset of 100 files from the archived dataset containing 1 14 733 ITS2 sequence files. These read benchmarks were designed to evaluate both sequential throughput for large data and random access efficiency for selective retrieval patterns typical in bioinformatics applications.

**Table 3:** Read performance statistics in milliseconds.

Metric		Pithos	MLA	HDF5	tar+gzip
Single large file	Mean	4,265	10,860	5,225	7,635
	Median	4,243	10,768	5,221	7,610
	StdDev	80	259	253	365
Random access	Mean	1,445	/	1,951	/
	Median	1,445	/	1,954	/
	StdDev	53	/	25	/
Sample large file pool	Mean	228	42,241	115	135
	Median	228	42,119	111	135
	StdDev	4	680	10	2

In addition to performance metrics, the evaluation examined the storage efficiency of archives created during the write performance tests to assess the disk space requirements of each solution (Table 4) across the different dataset categories.

**Table 4:** Disk usage of the create archives in bytes.

Metric	Pithos	MLA	HDF5	tar+gzip
Single large file	1,038,807,098	1,006,599,147	1,018,545,526	1,002,989,465
Multiple large files	1,142,635,165	2,013,696,632	2,037,195,870	2,006,129,961
Directory structure	181,087,574	174,583,864	190,280,741	180,533,076
Large pool of small files	46,490,778	16,478,528	343,034,270	14,411,674

The performance evaluation results show that Pithos performs better in most benchmark scenarios, especially in handling large files and reading operations. For single large file operations, Pithos achieved comparable write performance to HDF5, while both significantly outperformed MLA by approximately six-fold and tar+gzip by more than fourteen-fold. This extended to multiple large files, where Pithos now achieved write speeds definitely faster than HDF5’s, while MLA required more than seven times longer and tar+gzip took over 20 times as long. Pithos also delivers adequate performance in directory structure handling speed, again roughly equivalent to HDF5, but approximately two and a half times faster than MLA and over four times faster than tar+gzip. However, Pithos showed notably worse performance for vast amounts of small files, where MLA and tar+gzip demonstrated very high efficiency. This can be attributed to Pithos’s approach of compressing and encrypting each input file individually, which inevitably introduces computational overhead.

Notably, Pithos also demonstrated remarkable read performance advantages, operating distinctively faster than HDF and over twice as fast as MLA for full file extraction. For random access capabilities, Pithos again outperformed HDF5, while MLA did not provide random access via its command line interface and tar+gzip lacked this functionality entirely. Considering storage efficiency, Pithos demonstrated competitive compression ratios while maintaining its performance advantages. Standing out is the large file scenarios of very similar files where the Pithos archive did not grow significantly and only uses half of the disk space in comparison. MLA and tar+gzip achieved remarkably superior compression for small file collections.

## 5 Application

In the context of interdisciplinary research initiatives such as NFDI4Microbiota, which seeks to establish a consistent path from sequencing centers and other data-generating facilities to analysis and publication, Pithos provides clear advantages for data handling and integration. Traditional workflows in such settings often require multiple stages of compression, encryption, and re-encryption as data moves between institutions, each step introducing latency and potential vulnerabilities. By consolidating these processes into a single pass, Pithos produces archives that are immediately usable by downstream partners, reducing operational overhead and minimizing the risk of workflow fragmentation.

A central challenge for these initiatives lies in managing data access across a consortium of partners with different responsibilities and legal constraints. Pithos addresses this through block-level encryption that enables fine-grained access control. Sensitive clinical data can remain restricted to authorized partners, while de-identified sequence data is shared more broadly with analytical groups. This selective sharing supports compliance with data protection regulations without hindering collaborative efficiency, thereby facilitating cross-institutional research while safeguarding patient privacy.

Equally important is the ability to handle large datasets efficiently in cloud-native environments. Pithos provides random access to compressed and encrypted data, enabling researchers to retrieve specific genomic regions or subsets without downloading entire archives. This reduces bandwidth usage, accelerates interactive analyses, and allows shared cloud resources to be used more effectively. By embedding semantic metadata directly within archives, Pithos further ensures that contextual information such as sequencing parameters, quality scores, and analysis provenance remain permanently linked to the data itself. The schema-agnostic design allows each participant of the research community to maintain their established practices and validation tooling without requiring modifications to the container format itself. For instance, a metagenomics project might combine raw sequencing reads with MIxS-compliant metadata describing environmental samples and extraction protocols, attach Bioschemas annotations to downstream analysis results describing computational workflows and software versions, and finally package the entire research compendium with RO-Crate metadata when archiving to a public repository. Rather than enforcing a specific set of metadata schemata Semantic validation remains the responsibility of community-specific tools or platforms that integrate Pithos, ensuring that domain expertise drives the quality control. This capability directly supports the FAIR principles that are central to the scientific community mission, strengthening long-term reproducibility and interoperability across disciplines.

Finally, the append-only architecture of Pithos results in transparent audit trails that capture the complete history of data transformations from raw sequences to publication-ready outputs. Combined with storage optimization through content-defined chunking and deduplication, this approach reduces redundancy across overlapping datasets common in microbiome research while lowering storage costs. Together, these features make Pithos a powerful enabler for integrative bioinformatics, providing a consistent, secure, and efficient framework that supports collaborative discovery from data generation to publication.

## 6 Discussion

Pithos addresses long-standing challenges in research data management by integrating compression, encryption, metadata handling, and random access into an efficient container format. The results demonstrate that this approach benefits for typical life science scenarios. In particular, Pithos achieves consistently strong write and read performance for large and heterogeneous datasets while preserving efficient storage use. By embedding semantic metadata and providing an append-only audit trail, it further strengthens the reproducibility and interoperability of research data in alignment with the FAIR principles.

These findings have notable implications for collaborative research environments, where balancing data security, accessibility, and sustainability is crucial. Pithos allows researchers to manage large genomic and multi-omics datasets while maintaining fine-grained access and strong encryption. This capability is valuable for multi-institutional collaborations where different stakeholders may require controlled access to overlapping, yet not identical, subsets of data. Pithos’s integration of smart compression and content-defined deduplication shows its potential to reduce storage costs in cloud environments. These environments are becoming the default infrastructure for many scientific disciplines.

Nevertheless, certain limitations of Pithos must be acknowledged. The performance evaluation revealed that handling very large numbers of small files remains a challenge. In this specific case MLA and traditional archive formats performed more efficiently, but with the caveat that these archives are either encrypted in its entirety or not at all. Optimizing this aspect is still essential for broader adoption in fields where file collections with high item counts are common. Another important consideration is the barrier to adoption inherent in introducing a new container format. Widespread acceptance will depend not only on technical advantages but also on integration into existing tools, pipelines, and community standards. A key aspect of Pithos design is that it does not have to be adopted by a specific target group or a community as a whole, but can also be used transparently within backend infrastructures, for example as an internal storage optimization layer, without requiring users to interact with the format directly. This allows for the format to be integrated incrementally with existing storage backends and analysis workflows, minimizing disruption for researchers.

Future work will therefore focus on targeted improvements in three directions. First, optimization strategies for accelerating the ingestion of vast numbers of small files will be explored. On the one hand, the parallelized processing of input files and their blocks in order to better distribute the computational costs. On the other hand, a more efficient write buffer strategy will be evaluated to improve the number of I/O operations against the underlying storage. The current reference implementation has placed a stronger focus on consistency, whereby each file is written directly upon successful processing which causes a massive overhead in this scenario. Second, new tooling will be developed to enable direct interaction with storage backends, allowing Pithos archives directly to be written and read to and from different storage types. Third, mechanisms for efficient seek operations in compressed and encrypted archives will be refined, further enhancing the interoperability with analysis workflows and improving the user experience in large-scale cloud deployments. Taken together, these developments underline the potential of Pithos as a secure, efficient, and FAIR-compliant solution for research data management. By addressing both performance and semantic integration within a single format, Pithos provides a robust foundation for the future of collaborative, data-driven discovery in the life sciences.
